# Structuring of electrorheological fluids in polymer matrices for miniature actuators

**DOI:** 10.1016/j.heliyon.2024.e39138

**Published:** 2024-10-10

**Authors:** Jana Ihrens, Kathrin Marina Eckert, Irina Smirnova, Thorsten A. Kern

**Affiliations:** a Hamburg University of Technology, Institute for Mechatronics in Mechanics, Eissendorfer Str. 38, Hamburg 21073, Germany; b Hamburg University of Technology, Institute of Thermal Separation Processes, Eissendorfer Str. 38, Hamburg 21073, Germany

**Keywords:** Actuators, Braille, Tactile display, Oleogels, Lyogels, Electrorheological fluids, Miniature actuator

## Abstract

Miniature actuators are utilized in various application fields, from robotics to medical devices, where compact dimensions, precise movements, and cost-effectiveness are crucial factors. Particularly for applications like braille displays, there is a critical demand for lightweight, portable, and affordable actuators to integrate into daily life for visually impaired people. However, existing actuation technologies such as electroactive polymers, electrorheological materials, and piezoelectric elements often do not meet the specific requirements of miniature actuators, especially for braille displays. Therefore, this study investigates the behavior of electrorheological fluids incorporated into polymer matrices of cellulose and proteins for miniature actuators to overcome the primary challenge of sedimentation through the structuring of the liquid. The gel formulations are tested in three distinct setups: the V-shaped, horizontal plates, and valve system. These experiments demonstrated immediate structural changes in the gel formulations, achieving reversible movement. Furthermore, the valve setup even enabled the analysis of the strength of the hardened mixtures by the resistance against applied air pressure. The results demonstrate that incorporating electrorheological fluids into polymer matrices is not only feasible but also preserves the characteristic behavior of electrorheological fluids under electrical field exposure. This behavior validates the applicability and suitability of modified electrorheological fluid mixtures in miniature actuators.

## Highlights

• Electrorheological fluids can be used in polymer matrices for the application in miniature actuators.
• Sedimentation and toxic compounds can be avoided by using eco-friendly oleogels in electrostatic actuators.
• Development of scalable miniature actuator setup with potential suitability for multi-line braille displays.

## Graphical abstract

**Figure:**
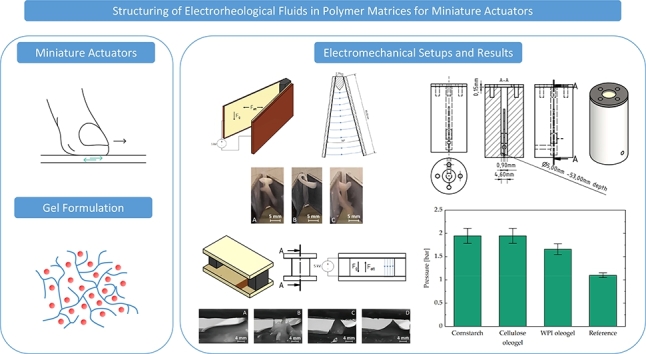

## 1 Introduction

Miniature actuators are relevant for various applications from robotics to medical devices, including braille or other high resolution tactile displays [1], remote haptic inspection systems [2] and rehabilitation robots [3]. These applications require devices with dimensions typically less than 10 cm edge length, capable of total displacement in the millimeter range, and accuracy often in the micrometer range. There are few actuators that can meet all these specific requirements, fitting into limited installation spaces while remaining affordable.

For braille applications, these limitations present significant challenges. Lightweight and portable braille displays are crucial for enabling visually impaired individuals to participate and communicate effectively in the digital world. These devices are essential for literacy and ensure social participation [4]. Braille displays, in particular, face high demands in terms of usability for multi-line braille arrays, compact size and weight, low power consumption, and overall affordability [4]. Previously explored approaches include the functional principles of electroactive polymers (EAPs), electrorheological materials, piezoelectric materials, and temperature-pneumatic actuation of fluids [5]. However, these functional principles often have drawbacks such as high costs, inability to be used effectively in braille arrays, and limitations in dimensions, weight, and power consumption, which reduce the portability and usability of corresponding devices for visually impaired individuals [5].

Various approaches applying valves in refreshable braille displays have been presented [4], [5], [6], [7], [8]. However, these approaches often encounter issues such as sedimentation of the electrorheological fluid (ERF) [6], [7], and serious consequences of leakage. Additionally, common formulations of ERFs frequently contain non-ecological components such as mineral or silicone oils, esters, or hydrocarbons, along with filler materials like silicon dioxide, titanium dioxide, or similar substances [9]. Nevertheless, ERF actuators offer distinct advantages for tactile displays, including low power consumption, the feasibility of matrix setups in confined spaces, and potentially low cost and reduced susceptibility to mechanical wear [10].

In this paper, ecological gel alternatives to conventional ERF mixtures are developed and tested in various setups. Unlike other studies [9], [11], where polymer components are directly added as filler material to ERFs, we incorporate ERFs into a porous polymer matrix. This novel approach of using the ERF in gel form proves effective in preventing leakage in portable devices and in structuring fluids to mitigate severe sedimentation issues [12], while also preserving the characteristic properties of ERFs.

In section 2, the state of the art for existing actuator concepts is discussed, highlighting the advantages and disadvantages of different approaches. Section 3 presents electro-mechanical setups using ecological formulations of ERFs in polymer matrices, followed by the gel formulation in Section 4. Additionally, Section 5 discusses the experimental results, with conclusions provided in Section 6.

## 2 State of the art

This section provides a comprehensive overview of relevant actuators, focusing on their applications, advantages, and key characteristics. This overview sets the stage for a detailed exploration of the interaction between ERFs and polymer matrices in miniature actuators. It emphasizes the significance in the development of miniature actuators, such as braille actuators and tactile displays.

### 2.1 Actuator overview

Actuators can generate force, torque, or displacement [13] using an energy source. Various physical principles can be employed for actuation, each suited to specific applications based on factors like power density, required forces, geometry, and refresh rates [14]. Below, we present the most commonly used physical principles and their respective advantages and disadvantages in haptic devices.

#### 2.1.1 Piezoelectric

Piezoelectric actuators utilize piezo crystals that deform physically under an applied electric field [15]. They are applicable in various configurations, including stacked, laminar, tubular, extender, or hybrid designs [15], [16], as well as piezoelectric bimorphs or piezo motors [15], [14].

Bimorphs consist of long, flat bending elements coated with piezoelectric material. When voltage is applied, one layer contracts while the other expands, causing the element to bend. Piezo motors, on the other hand, utilize piezo elements that are sequentially activated to enable rotation or translation. Key advantages of piezoelectric actuators include high refresh rates, low power consumption, and mechanical durability. However, despite these advantages, their potential applications are restricted by their large overall dimensions and costs, susceptibility to thermal variations, and limited displacement.

#### 2.1.2 Electromagnetic

Electromagnetic actuators function as force sources by utilizing magnetic energy to induce motion [14]. They encompass various types, such as translational, reluctance, and switching actuators, with rotary electric motors or stepper motors being commonly employed. These actuators are valued for their ease of manufacturing, robustness, and ability to generate high forces. However, electromagnetic actuators typically exhibit less dynamic characteristics and occupy a relatively large volume, which poses challenges for dense packing in matrices.

#### 2.1.3 Electrodynamic

Electrodynamic actuators operate based on the Lorentz force principle: a magnetic field generated by a permanent magnet interacts with a current passing through, resulting in a force perpendicular to both the magnetic field lines and the current direction. This technology is widely used in haptic applications with impedance control, as its output is directly proportional to the input [14]. Electrodynamic actuators can achieve rotary or translational actuation and are commonly employed as oscillators. However, electrodynamic actuators are known for their high power loss due to heat dissipation.

#### 2.1.4 Thermal

Several types of actuators exploit the effects of temperature changes, including thermo-pneumatic actuators and shape memory materials [14]. In general, temperature changes can influence mechanical properties such as stiffness or volume, which can be harnessed for actuation, although they typically have a slow response. Thermo-pneumatic actuators consist of a sealed chamber with one flexible side, filled with a low boiling point liquid. Raising the temperature with a heating element increases the pressure within the chamber, causing displacement of the flexible side. This process inherently requires high temperatures and thus high power consumption. Shape memory alloys can undergo reversible changes in shape. They return to their original state with an increase in temperature, switching between two crystal structures with small temperature changes [16].

#### 2.1.5 Electrostatic

Electrostatic actuators utilize the effects of static electric fields. Possible variants include capacitive actuators and electrochemical actuators [14]. Capacitive actuators consist of two oppositely charged conductive plates separated by an air gap [17], [14]. When a voltage is applied, a force is exerted on the plates, which can be directed in one direction by mechanical restraints on the plates. This type of actuator requires high voltages to achieve small displacements. However, its simple design and low power consumption are advantageous, and elastomeric dielectrics offer further design possibilities.

Electrochemical actuators can be divided into different subgroups. One of the major groups is solid-state actuators with EAPs. EAPs undergo volume changes when an electric field is applied, requiring a voltage in the kV range with low power consumption [14]. They can exert small forces with low dynamic properties.

Within the area of electroactive polymers, one can differentiate between dielectric and ionic EAPs [18]. Dielectric EAPs are characterized as electrostrictive materials capable of dimensional changes under an applied electric stimulus [19]. This category encompasses polymer groups such as dielectric elastomers, ferroelectric polymers, electrostrictive graft elastomers, and liquid crystal elastomers. These EAPs operate through the accumulation and interaction of opposite electric charges on the surfaces of elastomers. In contrast, ionic EAPs encompass ionic polymeric gels, conductive polymers, and ionic polymer-metal composites [18]. Unlike dielectric EAPs, the deformation of ionic EAPs is caused by the mobility or diffusion of ions in the polymer [18]. Compared to dielectric polymers, ionic EAPs require lower voltages to operate. However, the wetness of ionic EAPs must be maintained for their application as actuators [18]. In addition to EAPs, electrorheological fluids (ERFs) are a subgroup of electrochemical actuators [14] and are used as actuator materials in this study. ERFs exhibit an increase in viscosity under a sufficiently strong electric field [9]. Since they do not react with direct fluid deformation, additional mechanical energy must be supplied. ERFs have low power consumption and require small currents at high voltages in the kV range.

#### 2.1.6 Magnetorheological

The properties of a magnetorheological fluid (MRF), particularly viscosity, are influenced by magnetic fields as a result of minimizing the energy contained in a magnetic circuit [14], [20]. MRFs require low voltages but high currents to generate the magnetic field, whereas ERFs require high voltages but low currents. Disadvantages of MRFs include small displacement and challenging integration into matrix setups. However, MRFs require less volume than ERFs, exhibit highly dynamic behavior, and can generate large forces.

### 2.2 Comparison of actuator principles for the application

Depending on the application, various possibilities for miniature actuators based on different physical principles are presented. In the context of braille and tactile displays, criteria such as compact design, low-cost production, and high dynamics with low power consumption are crucial factors [21]. The concepts currently available for such applications are primarily based on piezoelectric bimorphs. Only a few approaches utilize rotating wheels or solenoids, which often offer less convenience. Most other actuator principles are nearly impossible to implement due to the restricted installation space required for multi-line displays. However, electrorheological applications offer a promising solution. The use of ERFs enables a denser arrangement of actuators for multi-line displays at a relatively low cost. Despite these promising prospects, several challenges remain, particularly the issue of ERF sedimentation, as identified in previous studies [6], [9]. Furthermore, ERFs raise environmental and safety concerns that must be addressed before they can be widely adopted and commercialized for tactile or braille displays.

### 2.3 Idea of ERF actuators

ERFs primarily consist of particles (heterogeneous) or liquids (homogeneous) dispersed in an insulating oil, potentially containing additives to further enhance their properties [22]. The application of ERFs for actuation utilizes their capability to alter rheological properties when exposed to an electric field [23]. These rheological changes are reversible and can be observed within milliseconds [24], [25]. Especially their fast response time enables the application of ERFs in various actuation processes, as proposed in studies focusing on medical robotics [26], haptics [27], or general actuation processes [28]. A primary challenge in the application of ERFs is the sedimentation of the dispersed phase over time [26]. Previous studies have used filler particles to improve sedimentation behavior [9]. Furthermore, current research has focused on porous polymeric filler materials to address sedimentation challenges, as demonstrated by Kuznetsov et al. [11], [29]. These studies have shown significant improvements in the sedimentation behavior of ERFs using natural polymeric filler materials.

In this study, we focus on porous materials. Instead of using aerogel particles as filler material, we incorporate the ERF into the polymer matrix, structuring it within the pores of the aerogel particles. This approach allows dispersed particles to remain mobile within the polymer matrix, thereby preventing sedimentation and maintaining the functionality of the mixtures over time. Structuring oils in polymer matrices draws upon previous studies in food industry processes [12], [30], [31]. Furthermore, this method offers ecological advantages compared to common ERFs, which typically consist of silicone or mineral oils with polyanilines, aluminosilicates, or other components [22], [9].

## 3 Electromechanical setup

Three setups were developed to analyze different gel formulations. The primary testing option utilizes a V-shaped plate setup, allowing for the adjustment of the distance between the plates for gel insertion. The gel formulations that respond in this setup were then tested in the second horizontal plate setup. Finally, a valve setup is use the applied force in practical applications. All setups were used with a *PHYWE* high voltage power supply, capable of delivering 0 to 10 kV with a maximum current of 2 mA to provide the necessary voltage.

### 3.1 V-shaped setup

**Figure 1:**
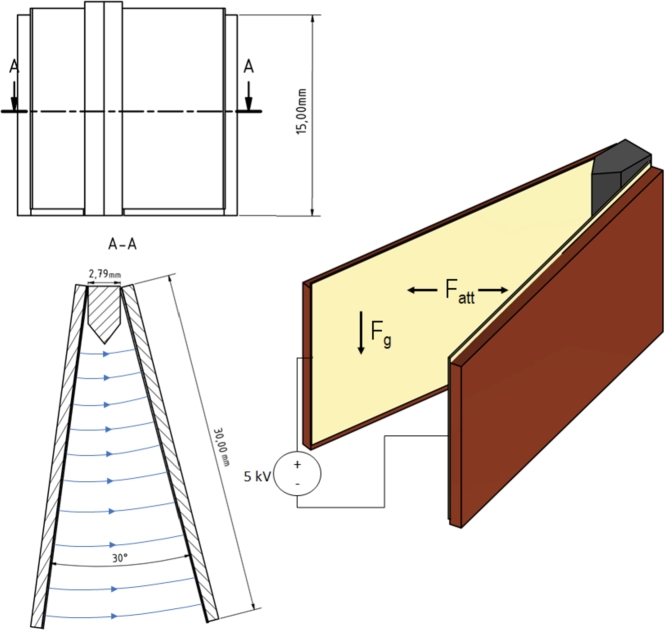
V-shaped Setup with Field Lines (blue).

The V-shaped setup was used to insert different gel formulations between capacitor plates with adjustable plate spacings. The setup consists of two plates with conductive copper coating covered with an insulating acryl resin varnish. This resign was applied as spray coating in a thin film on the equipment. Tests conducted with the V-shaped setup aimed to preliminary evaluate the response of the ERF formulations to an electric field. The setup is shown in Fig. 1. Connections to the high voltage power source were established by cables soldered to the ends of the plates with the larger spacing. The plates are separated by insulating tape at their closer edge and installed at an angle of 30°. Experiments were conducted with a voltage of 5 kV applied between the two plates. The different gel formulations were applied using a pipette from 2 mm above the setup's upper edge. The electric field strength of 0.63 ±0.05 kV/mm in the V-shaped setup corresponds to the outer edge of the setup. For the inner edge, where no droplets could be placed as it is too close in comparison to the droplet size, the field corresponded to 2 ±0.05 kV/mm. For the part that is wide enough to place the droplets, values from 0.63 ±0.05 to 1 ±0.05 kV/mm are achieved. The small distance at the V-point is due to manufacturing limits, higher V-point distances resulted in mechanical instability of the setup.

### 3.2 Horizontal plate setup

**Figure 2:**
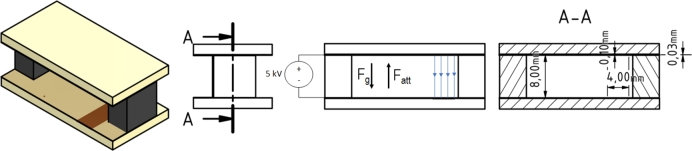
Plate Setup with Field Lines (blue).

A horizontal plate setup shown in Fig. 2 was used to test the reaction strength and reversibility duration of the gel formulation. Two printed circuit boards (PCBs), each 30 mm long and 8 mm high, were used, with a 4 by 8 mm copper area on opposite sides. To minimize the risk of arching between the plates, the PCBs were coated with insulating varnish, and the cable connections were made on the back of the PCBs using through-holes and pins at different distances from the copper surface to ensure sufficient spacing between the solder joints. The PCBs are separated by 3 mm thick insulating plastic blocks on either side of the setup. As in the V-shaped setup, the different gel formulations were applied with a pipette. High voltage was applied after the gel was inserted. In this setup, an electric field strength of 0.77 ±0.06 kV/mm was achieved.

### 3.3 Valve setup

**Figure 3:**
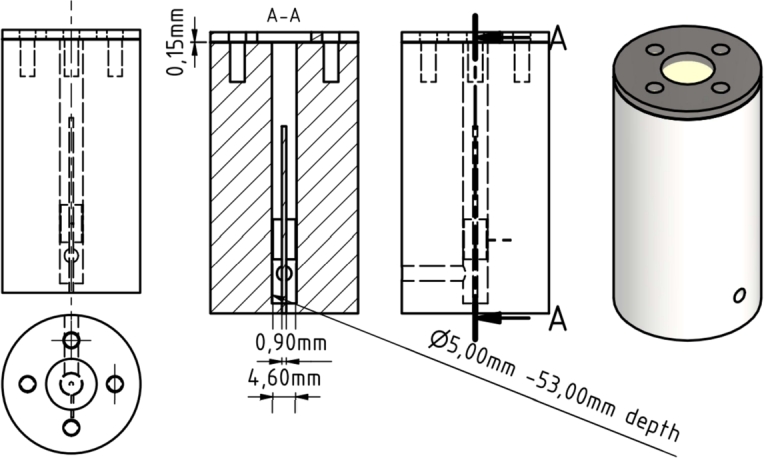
Valve Setup with Dimensions.

**Figure 4:**
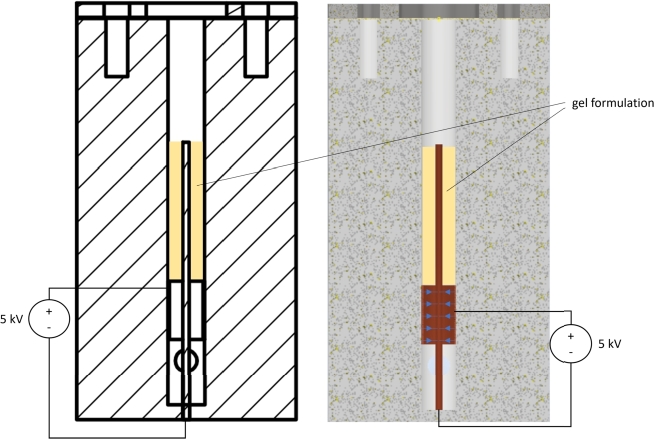
Valve Setup with Gel Formulation (yellow) and Field Lines (blue).

The experiments conducted with the horizontal plate setup and small spacers have revealed a significant influence on the required plate spacing. This effect, which may be attributed to slow discharging, capillary forces or other external influences, was considered in the development of a new specific setup. The setup utilizes a valve that is twice the size required for a braille display, as illustrated in Fig. 3. The setup consists of a vertical plastic cylinder with a central vertical hole housing a concentrically positioned copper pin. A horizontal connection to the central hole is made at the bottom to apply pressure. Above this connection, a conductive copper ring is positioned around the central pin at a distance of 1 mm. Cables for applying a high voltage of 3 to 5 kV between the conductive ring and the pin are led through to the outer side of the plastic cylinder, where connection plugs for further cabling are installed. All conductive surfaces are coated with insulating varnish. The central hole was filled with the gel formulation up to the top of the pin before attaching the membrane, as indicated in Fig. 4. During and after the filling process, the pressure application channel must remain closed. A 0.15 mm nitrile membrane is used to cover the top of the cylinder and is secured to the cylinder with a metal ring and four screws. In the valve setup, an electric field strength of 1.67 ±0.05 kV/mm was reached.

When filled with gel and covered by the membrane, the gel does not block the pressure applied by a syringe through the horizontal opening, thus lifting the membrane at the top. However, when a high voltage between 3 and 5 kV was applied, the gel accumulated and solidified between the conductive ring and the pin, effectively blocking the air pressure from lifting the membrane.

## 4 Gel formulation and characterization

Due to the integration of ERF into polymer matrices, this study diverges from existing literature, which predominantly focuses on exploring direct applications of ERFs themselves. In this study, polymeric gels serve as the matrix for structuring the ERFs. These gels are defined as cross-linked polymers or oligomers forming a convoluted network [32]. The porous structure of these polymer matrices enables the absorption of the ERF mixture into their pores [30]. Various ERF polymer formulations were tested and applied in different setups detailed in Section 3. Furthermore, this study prioritizes the use of sustainable materials and employs biopolymer approaches.

### 4.1 Aerogel production

The precursor materials of the considered gel types are cellulose (microcrystalline cellulose type II, JRS Pharma GmbH & Co. KG, Vivapure®) and whey protein isolate powder (WPI, BiPRO 9500 from Agropur, Longueuil, Canada). The synthesis of WPI and cellulose aerogel particles followed the procedures outlined by Jung et al. [30] and Schroeter et al. [33]. WPI microparticles were synthesized through a wet-milling step of the alcogels, while dried-milling was employed for the preparation of cellulose microparticles.

#### 4.1.1 Hydrogel synthesis

Whey Protein Isolate (WPI) powder was solubilized in deionized water at room temperature to create a 20 wt% solution. The solution was homogenized using an overhead stirrer for 3 h at 250 rpm. Subsequently, a mixture consisting of the protein solution and rapeseed oil (Henry Lamotte Oils GmbH, Bremen, Germany) with a weight fraction of 1:3 was prepared. The oil mixture was emulsified in a colloid mill (IKA Magic LAB 1508, Staufen i. Breisgau, Germany) in two cycles (rotation speed = 22,000 rpm, gap width = 600 μm. The WPI lyogel particles were formed in a beaker under stirring (10 min, 250 rpm) at 85 °C, using heat-induced gelation. The gel particles were separated from the oil using a 250 μm sieve and washed with deionized water.

Cellulose hydrogels were prepared using acid-induced gelation. The dissolution of cellulose was performed in two steps. Initially, 30 g cellulose powder was dispersed in deionized water. For swelling of the cellulose, the solution was kept at 5 °C for 30 minutes. Subsequently, the mixture was added to 270 g of a precooled 14 wt% NaOH (Merck KGaA) solution and stirred (500 rpm) at -9 °C. Cellulose particles were produced using the dropwise method by dripping the cellulose mixture into a 30 wt% aqueous sulfuric acid solution (H_2_SO_4_, Merck Millipore, Emsure®).

The obtained hydrogel particles from both precursor materials were washed with deionized water and transferred to the solvent exchange.

#### 4.1.2 Solvent exchange, milling and supercritical drying

A direct solvent exchange of the hydrogel particles to pure ethanol (EtOH, Carl Roth, 99.9 wt%) was performed. The solution was stirred for a minimum of 12 hs, and this process was repeated until the ethanol concentration reached 98 wt% in the liquid phase. The ethanol concentration was analyzed using density measurements (Anton Paar, DMA 4500 Graz, Austria).

The WPI particle production required an additional wet-milling step in the colloid mill (rotational speed = 22,000 rpm, gap width = 900 μm). During this step, the alcogel particles were dispersed in ethanol at a particle-ethanol ratio of approximately 1:10 vol%. The microparticles were collected after a brief sedimentation period through filtration using filter paper bags.

The prepared alcogel particles of cellulose and WPI were sealed into filter paper bags and subjected to supercritical drying in CO_2_. The drying process was carried out for 4 h in an autoclave with a volume of 4 L at 120 bar and 60 °C. The CO_2_ flow was set to 80-120 g/min, and a slow depressurization was employed (approximately 1 bar/min).

For the cellulose particles, dried-milling in the tube mill (IKA Tube Mill control) was performed after the drying step (rotational speed = 22,000 rpm, gap width = 900 μm).

The supercritical drying process enables the preservation of the porous network during drying, maintaining the pre-formed porous structures in the mesoporous region. Detailed investigations of the properties of the utilized aerogel particles can be found in the studies by Schroeter et al. [33] and Jung et al. [30], where the porous structure, pore sizes, firmness depending on oil-to-particle-ratio, and other properties are analyzed.

### 4.2 Oleogel preparation

According to the proposed method from Plazzotta et al. [12], the oleogels were prepared by adding the oil compound to the aerogel particles. Using this procedure, mixtures containing 12 wt% aerogels were produced. The final oleogels were collected by centrifugation afterwards, to separate them from the residual oil phase. Instead of sunflower oil, a mixture containing 55 wt% cornstarch (Unilever, Brussels, Belgium) and 45% rapseed oil (Brökelmann+Co, Hamm, Germany) was used.

For the utilization of cellulose particles, a prior hydrophobization step was required. The hydrophobization was carried out using the chemical vapor deposition technique with the solvent hexadimethylsulfoxide at 120 °C.

### 4.3 Characterization of gel formulations

To characterize the gel formulations, a sedimentation study was performed. Each formulation was mixed and left to settle for 48 hours. During this period, the height of the sedimented particle layer (a) and the height of the liquid supernatant (b) were measured. Using these measurements, the sedimentation ratio (r) was calculated: r=a/(a+b). This method follows the approach described by Shin et al. [34] and Kovaleva et al. [35]. In addition to the sedimentation study, rheological measurements were performed. The results are included in the supplementary information (Fig. S1, Fig. S2).

## 5 Results

In this study, we examined sedimentation and rheological properties as well as the responses of different gel formulations exposed to an applied electric field. The responses of the gel formulations to the electric field are compared and analyzed in the setups presented in section 3.

### 5.1 Sedimentation study

**Figure 5:**
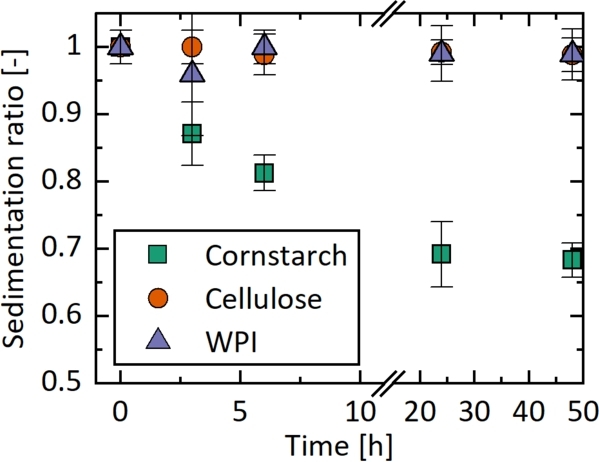
Sedimentation analysis over 48 hours. Comparison of cornstarch-oil (green squares), cellulose oleogels (orange circles), WPI oleogels (purple triangles). The error bars are 95 % confidence interval based on three measurements.

In the sedimentation study, the cornstarch-oil mixture without polymer incorporation, as well as formulations with oleogels from cellulose and WPI, were tested (Fig. 5). Comparing these materials, a significant improvement was achieved by incorporating the ERF into a polymer matrix.

Sedimentation of the cornstarch-oil mixture was visibly apparent after 3 hours and reached a minimum of 0.69 ±0.05 after 24 hours. In contrast, no sedimentation was observed during the experiments for the oleogel formulations. Even after 48 hours, no significant sedimentation was observed. Oleogels, as described in Section 4, are produced by absorbing oil into the porous polymer matrix. The absence of significant sedimentation observed in the analysis suggests that this oil remains within the porous structure of the polymers throughout the study. Compared to the established method of adding filler particles to ERFs [35], incorporating the ERF into a polymer matrix not only improves sedimentation behavior but also facilitates easier handling of the materials in electrical setups as the fluids are retained within the polymer matrix. This approach makes the materials easier to use in applications such as braille and tactile displays.

### 5.2 V-shaped setup

**Figure 6:**
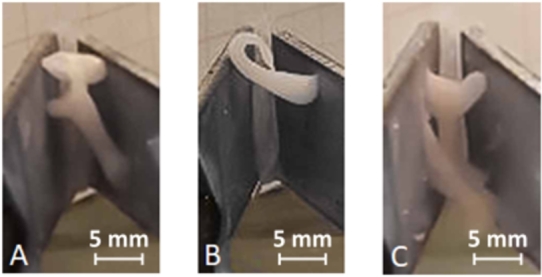
Performance of mixtures in V-shaped setup: Cornstarch-oil (A), cellulose oleogels (B), WPI oleogels (C).

In the initial phase of this study, a V-shaped setup was utilized to quantify the reactions of different gel types. In these experiments, the analyzed mixtures were dripped into the V-chamber and exposed to an electric field. It was observed that the initial contact of ERF with the electric field resulted in the immobilization of the mixture (Fig. 6).

As expected, the cornstarch-oil mixture was attracted to both plates of the V-shaped setup. This observation suggests that the cornstarch molecules undergo polarization in response to the electric field, resulting in attraction to both positive and negative poles. Consequently, the well-known ERF was immobilized, forming a bridge between the plates. This behavior was replicated with the aerogel mixtures of cellulose and WPI, indicating that the incorporation of the ERF into an additional polymer matrix does not alter the response behavior. Moreover, these mixtures exhibit a short response time, making them suitable for actuation systems. Compared to commercial ERFs, incorporating the ERF in the polymer matrix enhances their handling by structuring the oil mixture. Additionally, utilizing bio-polymer matrices with rapeseed oil offers an environmentally friendly alternative to synthetic approaches. In addition to the oleogel formulations containing cornstarch, pure cellulose oleogels were also investigated. Since cellulose is a known filler material for ERFs [35], a similar response to the cornstarch-oil mixture was expected. However, only minor attraction of the fluid was observed at the highest field strengths for small droplets. This reduced behavior of the material may be attributed to structural changes caused by supercritical drying, the absorption of oil into the oleogel's pores, or simply the inability of the solid cellulose matrix to respond to the electric field. Based on these findings, pure cellulose oleogels are not suitable for actuation purposes and are not further investigated in this work.

### 5.3 Horizontal plate setup

**Figure 7:**
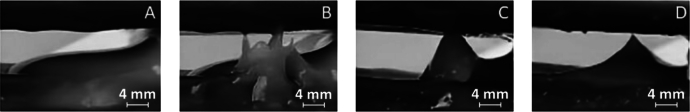
Performance of mixtures in horizontal setup. Without applied electric field: Cornstarch-oil mixture (A). With applied electric field: cornstarch-oil (B), cellulose oleogels (C), WPI oleogels (D).

In the next step, the mixtures were tested in the horizontal plate setup and compared to the previous results. Unlike the V-shaped setup, the horizontal orientation of the plates allows for analysis of the mixture's attraction strength to the plates. If the attraction force is too weak, the mixture remains unaffected; however, if the force is strong enough, the liquid is drawn up to form a bridge between the positive and negative poles (Fig. 7).

When an electric field is applied to the cornstarch-oil mixture, direct attraction occurs towards either the positive or negative pole. Due to the polarizability of cornstarch in this system, the attraction towards the opposite pole is strong enough to pull the liquid upward by 3 mm and hold it in place. Even after the electric field is turned off, the liquid remains in position, likely due to slow discharge or occurring capillary forces. This phenomenon is not observed in the V-shaped setup, suggesting it is a characteristic effect of the plate setup rather than the specific formulations being tested. The performance of the oleogel mixtures was less affected by the electric field, as it did not cause autonomous upward movement towards the opposite pole. Oleogel mixtures inherently have higher viscosity compared to the cornstarch solution, requiring a stronger attraction force to draw the liquid towards the upper pole. Initially, the attraction force was insufficient to move the liquid upward by the full 3 mm distance, but when the distance was reduced to less than 1 mm, autonomous upward movement was observed. This demonstrates that the electric field can induce movement in the oleogel mixtures as well; however, achieving a similar response as in the less viscous cornstarch solution requires a greater attraction force.

When comparing the effect of electrostatic discharge on the mixtures, the cornstarch solution responded directly to this change. The peaks not in direct contact with the upper pole immediately returned to the lower plate. In contrast, the larger peak bridging between the two plates remained in position even after discharge. Due to the small scale of the setup, this behavior is likely influenced by capillary forces rather than the electrorheological properties of the mixture. In experiments with the WPI mixture, this phenomenon was more pronounced, as the smaller peak connecting the plates was released immediately after discharge.

It can be concluded that ERF mixtures not only possess the capability to alter their rheological behavior but also demonstrate movement due to their polarizability. The mixtures are attracted to the poles when the electric field is applied, connecting and forming a bridge between them. This behavior can be effectively utilized to induce movement of the ERFs.

### 5.4 Valve setup

**Figure 8:**
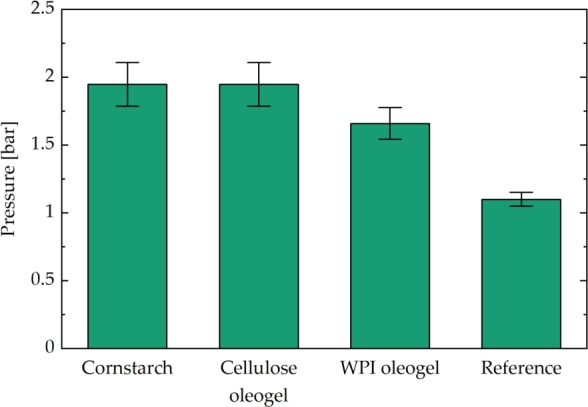
Maximum pressure reached in valve setup before breakthrough. The error bars were calculated for a measurement uncertainty of the injected volume of ±0.2 mL. The reference was analyzed with cornstarch mixture in the setup without the applied electric field.

The previous results highlight a wide range of potential applications for these systems. Particularly notable is their behavior in actuation processes. Therefore, this section focuses on a specific application: utilizing these systems as valves in braille or other small actuator systems. According to Fig. 3, the analyzed gel formulations were filled into the equipment. With no electric field applied, air flow through this fixed bed of mixtures can be achieved. However, activating the electric field is expected to close this channel due to the immobilization of the ERF mixtures. The air pressure applied at the bottom of the setup was generated through manual compression of a piston in a syringe. Due to the challenges associated with pressure measurements at the tested scale, the volume change in the syringe was measured to calculate the applied pressure using the ideal gas law. The maximum pressure that could be applied before air breakthrough, indicated by the membrane's extension, was investigated for all formulations (Fig. 8).

Starting with the cornstarch-oil mixture, a pressure of 1.95 bar ±0.16 bar could be applied to the valve before air breakthrough occurred. The performance of the cellulose oleogels was similar, whereas WPI oleogels enduring slightly lower applied pressure. As a reference, we analyzed the necessary pressure to achieve a 2 mm extension of the membrane without applying an electric field. These reference measurements demonstrated that minimal overpressure was sufficient to achieve the required membrane extension for braille applications. A comparison of the results between the cornstarch-oil and oleogel mixtures with the reference measurements strongly supports the potential application of ERF-derived mixtures as smart materials in actuation processes. As in the previously tested setups, the material responded immediately to the electric field after the discharge of the system. Moreover, the responses to charging and discharging could be successfully repeated.

For future applications, the membrane of the valve setup can be scaled down and used, for instance, to raise a pin as a Braille dot. By enclosing the polymer-containing region of the setup and sealing it tightly against liquids, the setup can offer a secure option for Braille actuation.

## 6 Conclusion

In this work, the applicability of ERFs for small actuation applications, such as in braille displays, was proven. For the first time, it was demonstrated that the incorporation of ERFs into polymer matrices is feasible and does not affect the response behavior of the ERFs. This approach not only improves the structural properties of the ERFs but also effectively addresses challenges associated with sedimentation. Furthermore, it presents a non-toxic and eco-friendly alternative for applications requiring small-scale actuation. Beside their environmental-friendliness, oleogels are comparably easy to manufacture and could provide a cost-effective solution for various types of electrostatic actuators. Considering this, new design-principles for actuators can emerge, utilizing large reservoirs of oleogels to operate in large-scale distributed arrays, such as in braille displays, or for more robust industrial applications in the future. Combining them with pneumatics, hydraulics, or other traditional actuation principles will enable reconfigurable mechanical impedances, extending even into the realm of soft robotics.

## Funding

This project is funded by the 10.13039/501100001659Deutsche Forschungsgemeinschaft (DFG, German Research Foundation) – SFB 1615 – 503850735. Jana Ihrens and Kathrin Marina Eckert acknowledge the $i^{3}$-Junior Project funding, which is an internal research funding program at 10.13039/501100023890Hamburg University of Technology (TUHH). Publishing fees supported by Funding Programme Open Access Publishing of 10.13039/501100023890Hamburg University of Technology (TUHH).

## CRediT authorship contribution statement

**Jana Ihrens:** Writing – original draft, Visualization, Project administration, Methodology, Investigation, Funding acquisition, Conceptualization. **Kathrin Marina Eckert:** Writing – original draft, Visualization, Methodology, Investigation, Funding acquisition, Conceptualization. **Irina Smirnova:** Writing – review & editing, Supervision. **Thorsten A. Kern:** Writing – review & editing, Supervision.

## Declaration of Competing Interest

The authors declare that they have no known competing financial interests or personal relationships that could have appeared to influence the work reported in this paper.

## Appendix A Supplementary material

MMC Supplementary information: Structuring of Electrorheological Fluids in Polymer Matrices for Miniature Actuators MMC
