# Supplementary material for: Structuring of electrorheological fluids in polymer matrices for miniature actuators
Source: Heliyon. 2024 Oct 10;10(20):e39138. doi: 10.1016/j.heliyon.2024.e39138 (PMC11620072; doi:10.1016/j.heliyon.2024.e39138)
Supplement: MMC — Supplementary information: Structuring of Electrorheological Fluids in Polymer Matrices for Miniature Actuators [file mmc1.docx]

**Structuring of Electrorheological Fluids in Polymer Matrices for Miniature Actuators**

Jana Ihrens^a^, Kathrin Marina Eckert^b^*, Irina Smirnova^b^, Thorsten A. Kern^a^

a] Institute for Mechatronics in Mechanics
Hamburg University of Technology

Eißendorfer Straße 38, 21073 Hamburg, Germany

b] Institute of Thermal Separation Processes

Hamburg University of Technology

Eißendorfer Straße 38, 21073 Hamburg, Germany

**Rheological Characterization of Oleogel Formulations**

The rheology measurements were performed with a Malvern Kinexus Pro rheometer (KNX 2100, Malvern Instruments GmbH, Herrenberg, Germany) using an oscillating plate-plate geometry (PL65 S 3185 SS and CP4/40 SR5206 SS).


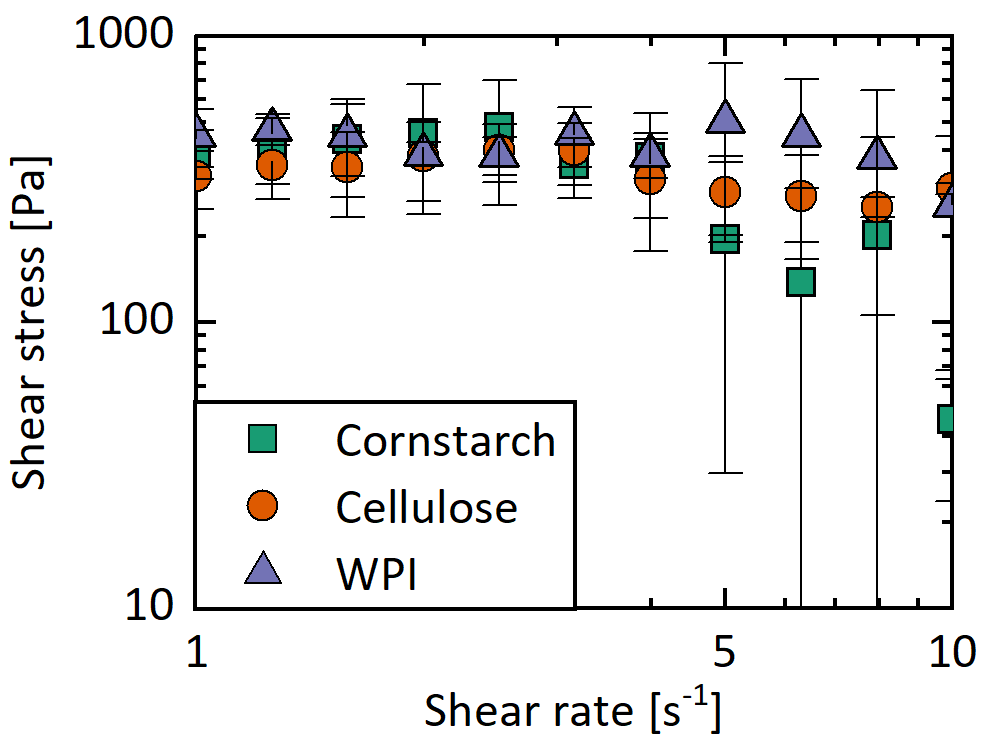


Figure S 1. Shear stress vs. shear rate of the prepared formulations without the application of an electrical field. The standard deviations are calculated based on multiple experiments (n=3).


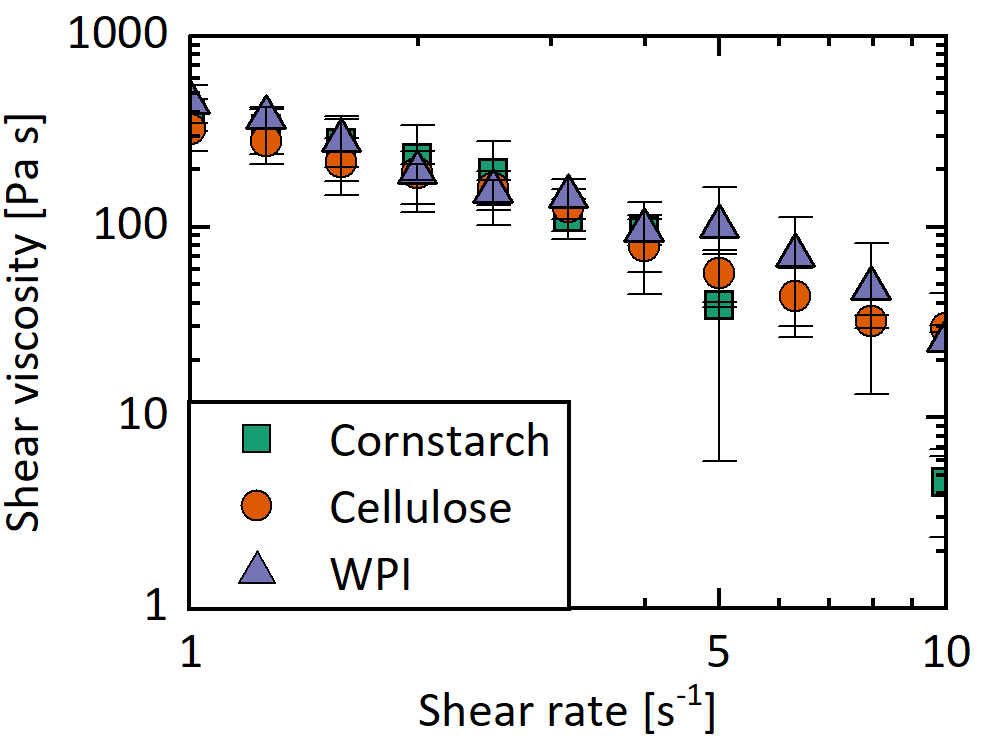


Figure S 2. Shear viscosity vs shear rate of the prepared formulations without the application of an electrical field. The standard deviations are calculated based on multiple experiments (n=3).
